# ‘Drowned in a Sea of Inhumanity’: Natural Childbirth, Postnatal Depression and the National Childbirth Trust, 1956–80s

**DOI:** 10.1093/shm/hkad083

**Published:** 2023-10-31

**Authors:** Hilary Marland

**Affiliations:** Department of History, University of Warwick, Coventry, CV4 7AL, UK

**Keywords:** National Childbirth Trust, postnatal depression, natural childbirth, experts, feminist health movement

## Abstract

During the 1970s, the National Childbirth Trust (NCT) began to provide information and support to women experiencing postnatal mental illness, building on its promotion of natural childbirth and emphasis on the emotional wellbeing of women around birth, which had occupied the organisation since its establishment in 1956. This article argues that, alongside emotional, social and medical factors, the NCT attributed postnatal depression to the shift to hospital deliveries, involving high levels of intervention and frustrating women’s choice and agency. While sharing ambitions to improve care in childbirth and giving women a voice in describing their experiences, it is suggested that the NCT’s relationship with the feminist health movement remained ambiguous. The article also explores the NCT’s collaboration with a variety of experts and advisors, some of whom emphasised the risk of postnatal depression to the bonding process and infant’s development, potentially exacerbating the mental distress of new mothers.

During the 1970s, the National Childbirth Trust (NCT) began to offer support to women experiencing postnatal mental illness, in response to what the organisation saw as a lack of information and practical assistance for mothers confronting debilitating and potentially long-term mental illness after giving birth. This coincided with growing interest in postnatal depression in the medical press, the media and amongst mothers and their families. Utilising its postnatal classes and support groups, study days, conferences and publications, the focus on postnatal depression, and, to a lesser extent, the more severe but much rarer condition of postpartum psychosis, represented a new initiative for the NCT. However, this article will argue that this built on longer-term interest in the emotional impact of pregnancy and childbirth that had captured the attention of the NCT since its foundation in the mid-1950s. The bedrock for a response to postnatal depression was established in connection with the NCT’s advocacy of natural childbirth and resistance to what it saw as invasive obstetrics in hospital settings that left women feeling powerless, isolated, anxious and depressed. Coinciding with the emergence of a wider cultural critique of medicine, the consumer health movement and women’s health activism, the NCT also aimed to open up conversations about postnatal mental illness, which would enable women to seek help and counter the stigma associated with mental breakdown in new mothers. Yet, as this article also argues, the NCT’s model of idealised natural birth and its highlighting of the risk of postnatal depression to the bonding process, may have contributed to rather than reduced mothers’ mental distress.

The NCT was one of several organisations and charities established in Britain over the course of the twentieth century to lobby for improved maternity services. At the beginning of the century the direction of maternity provision and childbirth as a demographic phenomenon was principally the preserve of politicians and doctors, and women were largely excluded from shaping their own services and care. However, increasingly they began to organise, lobby and campaign through a variety of professional, voluntary and political organisations, including the Women’s Cooperative Guild, the Labour Party Women’s Organisation, the Mothers Union, the National Council of Women and the National Birthday Trust Fund.^1^ Up until the Second World War campaigns focused on the urgent need to reduce maternal mortality, morbidity and suffering, the extension of training for midwives and obstetricians, and mitigating the impact of poverty on expectant mothers, amidst the national imperative to encourage healthy motherhood as a civic duty. After the 1930s maternal mortality dropped significantly, midwifery training and the education and regulation of obstetricians improved, local authority maternity services became more effective, maternity benefits were introduced, and in 1948, the NHS ushered in free childbirth services for all.^2^

In the post-war period newly established organisations, while still devoting attention to improving standards of maternity care, began to focus increasingly on rights and benefits and women’s individual childbirth experiences and choices. Significantly, this coincided with a major shift in the locus of birth in the post-war period, a shift which would become a key focus of NCT campaigns. In 1959, the Cranbrook Report called for 70 per cent of births to take place in hospital and the Peel Report of 1970 advised, largely on grounds of safety, that facilities should be set up to enable all women to give birth in hospital. Between 1963 and 1972, hospital deliveries rose from 68.2 per cent to 91.4 and after 1975 never dropped below 95 per cent.^3^ Yet, as birth moved increasingly from home to the hospital, this led to concerns about ‘inhumane’ conditions in many maternity units, while the 1950–80s also marked the heyday of the natural childbirth movement, which aimed to improve women’s experiences of birth and reduce their reliance on pain relief. These changes took place against a backdrop of profound changes in women’s lives, as many women moved into higher education or paid employment, and potentially experienced more reproductive and sexual autonomy.^4^ Second-wave feminism and the health activism of the 1970s and 80s, meanwhile, shaped many women’s views and experiences of sexuality, reproductive health and childbirth.^5^

Campaign organisations set up in the second half of the century reflected and responded to these major shifts in women’s lives and experiences of childbirth. In 1958, the Society for the Prevention of Cruelty to Pregnant Women was founded by Sally Willington, following her unhappy experience of hospital birth. Renamed the Association for Improvements in the Maternity Services (AIMS) in 1960, it campaigned energetically for better services and treatment of women giving birth in hospital, for fathers to be able attend deliveries, and to expand knowledge of childbirth among women and their families. By the 1980s, AIMS was lobbying for the right to home births on the basis that women should be able to decide where to give birth, and because research suggested that hospitals were no safer than home deliveries.^6^ In 1980, Maternity Alliance was established with the aims of reducing inequalities in childbirth services and improving maternity leave and benefits, and worked particularly closely with trade unions and groups who were poor or otherwise disadvantaged and lacked a voice in health care provision.^7^

However, it is the NCT that will be the focus of this article. Though its leadership and membership were predominantly middle class (with membership costing 5 shillings per annum in the late 1950s), and its reach thus limited, the NCT became the largest charity advocating for women in childbirth and early parenthood in post-war Britain. By 1962, the organisation had 19 branches, 39 by the early 1970s and 320 by 1986, when it had over 40,000 members.^8^ Describing itself as a ‘consumer council for childbirth’, as well as offering antenatal classes, postnatal support and practical information, the NCT functioned as a social network and a pressure group for women’s rights, contributing to policy debates and shaping maternity provision at a national level. Aiming to improve hospital care and to resist increasingly interventionalist practices, the NCT was particularly visible in its campaigns for access to home births and choice for women and their families, which were frequently reported in the national and local press. It was also influential. From its establishment in 1956, the NCT was led predominantly by middle-class women with good contacts. Alongside well-known birth activists, most notably founder member and NCT teacher Sheila Kitzinger, the NCT built close and fruitful relationships with doctors and later midwives and health visitors sympathetic to their approach and objectives.^9^ This meant, as Tania McIntosh has put it, that ‘things got done’ by the NCT.^10^ The NCT was thus in a strong position to raise awareness of postnatal depression amongst medical professionals and women and their families, and was able to engage a wide range of advisors and experts united behind the principle that increasingly medicalised births presented a risk to women’s emotional wellbeing and needed to be resisted. Importantly, the NCT also included women who had experienced postnatal mental illness as experts, who were regarded as well-placed to inform and advise other mothers.

## Natural Childbirth, Inhumane Obstetrics and the NCT

What was initially known as the Natural Childbirth Association (NCA) was established in 1956 by Prunella Briance after the tragic death of her baby following conventional obstetric care. Initially, the organisation focused on the promotion of Dr Grantly Dick-Read’s system of natural childbirth, and interest in the NCA grew rapidly, fuelled by women writing in with unhappy stories of childbirth and enthusiastic responses to Dick-Read’s teaching.^11^ Advocating simple exercises with an emphasis on breathing, making women fit for labour and giving them confidence and the ability to relax, Dick-Read claimed to equip women to overcome the emotion of fear and avoid what he termed the F-T-P (Fear-Tension-Pain) Syndrome.^12^ Women, Dick-Read argued, needed to be ‘trained’ to ‘discipline’ their emotions, and tactfully and carefully initiated into the job they were about to perform.^13^ With this psychological preparation and by building a close relationship with their birth attendants, labour would be shortened, pain reduced or eliminated and women might avoid instrumental or surgical intervention. Emphasising the naturalness of pregnancy and birth and resisting obstetric intervention and the move towards hospital deliveries, the natural childbirth movement was in some senses ‘radical’. Yet in its early decades it also embodied deeply conservative values. Dick-Read appealed directly to women’s sense of social responsibility and emphasised their roles as mothers and homemakers, an emphasis shared in the NCA’s highlighting of the importance of ‘happier births for the welfare of the nation’.^14^ It was largely well-resourced middle-class women who chose natural childbirth, an approach less likely to appeal to working-class mothers attracted by the option of pain relief and the respite offered by hospital births.^15^

In 1958, the organisation gained charitable status, becoming the Natural Childbirth Trust and in 1961, the National Childbirth Trust, ‘because it had been reported that doctors were offended by the word “natural”‘.^16^ By the early 1960s, the NCT had shifted its allegiance from Dick-Read to promote Fernand Lamaze’s teaching of ‘prepared childbirth’ or psychoprophylaxis. NCT teacher Erna Wright was particularly influential in introducing Lamaze’s approach, which supposed that childbirth was inherently painful but that by teaching relaxation and breathing techniques, and with women’s active participation and effort in labour, they could distract themselves from the pain.^17^ In advocating natural childbirth, the NCT emphasised the importance of emotional support and boosting women’s confidence in labour, arguing that the mother’s mind had been neglected in pregnancy and labour. Though both Dick-Read and Lamaze suggested that authority would remain largely with the physician during delivery, already in 1957 NCT officers were emphasising the importance of access to home births, where it was assumed that mothers would retain more control of their birth experiences.^18^

Natural childbirth techniques were introduced in small antenatal classes, led by women who had experienced childbirth and completed a six-month probationary training course. Though referred to as ‘lay teachers’, many had a background in midwifery, nursing or physiotherapy or had taught remedial exercises. Initially, the NCT trod a cautious path, carefully judging the tone of their appeals in encouraging natural childbirth while seeking to win the trust and cooperation of doctors and midwives, and women attending classes were urged to request written permission from their doctor or midwife.^19^ The NCT noted that their preference was for doctors and midwives to ‘participate in the mother’s training or better still take charge of it’, and saw their own role in this as eventually diminishing.^20^ Dr Stanley Perchard, NCT chairman in 1962, stressed that ‘the training should at all costs avoid dividing the mother’s loyalty or impairing her confidence in her professional attendants’.^21^ Notwithstanding this caution, natural childbirth was opposed by many doctors and midwives. Dr Ian Donald, who encouraged a scientific approach to obstetrics, claimed that Dick-Read had ‘developed almost a cult for the easing of childbirth’s miseries by mental as well as physical relaxation... All this huge and varied fabrication is based on the observation that the course of labour can be modified by the patient’s mental state.’^22^ Citing a NCT meeting where Lamaze’s work was promoted, he suggested that natural childbirth was redundant, and that psychological preparation was no more than that offered by the ‘competent humanity and care’ of a trained obstetrician and the essence of good doctoring.^23^

Other obstetricians, however, were less convinced about the direction of travel of maternity care, which as they put it, ‘dehumanised’ obstetrics, failed to account for mothers’ emotional needs and resulted in growing levels of maternal anxiety, stress and depression. A number of these specialists worked closely with the NCT. In 1960, while serving as an NCT advisor, Dr Norman Morris, an obstetrician based at Charing Cross Hospital in London, published a landmark article in *The Lancet*. He argued that, while progress had been made in reducing the physical risks of childbirth, serious gaps remained in terms of understanding ‘the patient’s emotional condition in pregnancy, labour, and the puerperium’.^24^

For most women childbirth is their moment of greatest achievement and sometimes of greatest happiness. It is an immensely important emotional as well as physical event... The influences that a woman meets during this time may have a tremendous psychological and social significance, and in my view our present hospital system often fails miserably in its care of the patients’ emotions. The joys, hope and wonder that the arrival of a new life should bring are spoiled and splintered into loneliness, indignity and despair. The feeling of personal achievement is lost, drowned in a sea of inhumanity.^25^

Morris’s views had been shaped not only by his own observations but also by reading extracts of letters sent to a weekly women’s journal, summarising the impact of badly run maternity units, which offered women little emotional support. He criticised ‘conveyer belt’ antenatal care, the lack of attention paid to emotional preparedness for child-birth, the loneliness, fear and poor, even rough or cruel, treatment that women might experience during delivery, and separation from the baby after the birth. One woman cited by Morris described how during labour her mental condition was ‘indescribable’. She was left alone for five hours, and afterwards was ‘robbed of my peace of mind for many months to come’.^26^ In 1961, the Ministry of Health responded with a short report ‘Human Relations in Obstetrics’, containing a series of recommendations intended to address the issues Morris and others had highlighted. The report also pointed out that mothers were now better informed and ‘will no longer blindly follow the advice given’.^27^

The report, however, changed little in terms of obstetric care in many hospitals, which by the mid-1960s saw childbirth as a highly managed and sterile procedure that should take place in the lithotomy position, with women on their backs and their feet in stirrups, the doctor and midwife donning gowns and masks as if for a surgical operation.^28^ Hospital maternity wards were accused by mothers of ‘inhumanity’ as they complained of being treated ‘like a cow in calf’ and about a lack of contact with their babies after the delivery.^29^ The NCT continued to lobby for improvements. At the same time, they began to claim success for what they had already achieved and in 1967, summarising the work of the NCT’s first decade, retiring Chair Lady Mickelthwait, confidently emphasised the value of the practical and emotional support offered through NCT classes and antenatal training, by

people who recognise the importance of mind and emotions and look to the effect they can have on the experience of childbirth. Today, women can look forward to the birth of their babies in a joyous fashion which bears no resemblance to the resignation of earlier generations. Women today want to know all about childbirth.^30^

As hospital birth became increasingly the norm by the 1970s, the NCT began to promote ‘a less accommodating approach to medical professionals’, and its campaigns focused increasingly on questioning interventionalist obstetrics, notably induction and caesarean section, as well as insisting on women’s right to choose a home delivery.^31^ These campaigns paralleled wider cultural critiques of medicine, associated in particular with Ivan Illich, who condemned processes of medicalisation, the direct harm done by medicine and the disempowerment of the patient.^32^ Having framed itself as a ‘consumer council’ for ‘expectant mothers’ since its establishment, the NCT also shared in an emerging and broadly based consumerist health movement, centred on demands for choice and patients’ rights.^33^ This was highlighted when Sheila Kitzinger published *The Good Birth Guide* in 1979, which assessed hospitals in terms of the quality of their antenatal care and treatment of women during delivery, based largely on information provided by mothers in response to requests placed in women’s magazines, *The Sunday Times* and *Evening Standard*.^34^ By this time images and descriptions of birth and criticisms of maternal care featured increasingly in public debates, the press and women’s magazines. In 1973, Margaret Allen challenged the move from home to hospital as a place of birth in an article published in *The Times*, describing her own experiences of a ‘horrifying’ hospital delivery that left her feeling ‘physically, mentally and emotionally assaulted’, and regretting the policy that virtually forbade women from having their babies at home.^35^

A decade later, the *Daily Mail* described ‘The Birth of a Revolution’ in maternity wards with women, working with organisations like the NCT, winning improved maternity care and more choice.^36^

By the 1970s, natural childbirth would also become closely bound up with feminist health movements and in some instances countercultural campaigns around childbirth.^37^ Emphasis shifted to empowering women and their partners, and resisting the ‘medicalisation’ of birth, giving women active roles which would enhance their emotional wellbeing and birth experiences. Yet with the issues identified by Morris unresolved and even exacerbated by the increased use of obstetric technology, the shortcomings of obstetric care in terms of women’s emotional experiences continued to be highlighted. It was against this backdrop that postnatal depression was identified as a prevalent and alarming condition for new mothers, which many observers related to women’s continuing lack of autonomy in childbirth and experience of hospital births.

## Framing Postnatal Mental Illness

The first half of the twentieth century was marked by intense debate among psychiatrists as to whether depression and psychosis related to pregnancy and childbirth existed as discrete disorders, and the labelling of mental illness linked to childbirth varied enormously. By 1900, the validity of the widely accepted diagnosis of ‘puerperal insanity’, first described in 1820 as a severe form of mental breakdown commencing within a few weeks of childbirth, was brought into question, and many psychiatrists argued that while pregnancy and birth might contribute to mental illness they did not warrant distinct diagnoses.^38^ Despite this, the term ‘puerperal insanity’ was still used in a number of mental hospitals and psychiatric and obstetric textbooks and the courtroom in trials involving the defence of new mothers in infanticide cases into the interwar period.^39^ Alongside this, looser descriptors were adopted, such as ‘insanities of reproduction’ or ‘psychoses associated with childbearing’ or women were diagnosed as suffering from more general psychiatric conditions, including acute mania, confusional or delusional insanity, or dementia praecox, with pregnancy and childbirth listed as associated causes.^40^ Despite this diagnostic confusion, there was sustained medical interest in mental illness related to childbirth during the late nineteenth and first half of the twentieth century.^41^ After the Second World War, however, mental disturbance among mothers took on new associations. As Sarah Crook has suggested, during this period medical professionals began to focus increasingly on the impact of the mother’s mental health on the family and particularly the newborn baby.^42^ In 1955, for example, A.B. Hegarty referred to the significance of what he termed ‘post-puerperal depression’ for its frequent occurrence and for the distress it caused both the patient and the family group.^43^ While the risk of infanticide had been consistently emphasised since the nineteenth century, after the 1950s, a broader range of anxieties were highlighted, focusing on less extreme but nonetheless pressing fears about maternal attachment, child development and neglect, with the mother’s mental illness depicted as an impediment to bonding.

Towards the end of the 1960s, there was growing interest amongst the medical profession, the media and women themselves in what was labelled puerperal depression, and by the 1970s more typically postnatal depression. In 1968, after being alerted by a health visitor in the East End of London to the prevalence of depression in women who had recently given birth, psychiatrist Brice Pitt provided evidence on what he regarded as a common and serious form of postnatal mental illness, ‘that lies between the extremes of severe puerperal depression... and the trivial weepiness of “the Blues”‘.^44^Pitt also pointed to a lack of medical knowledge about what he considered to be an important complication after birth, and sought to establish diagnostic order (though many psychiatrists would refine or dispute his definitions) in his re-formulation of the categories of maternal mental illness as psychotic depression, puerperal depression and the ‘baby blues’.^45^ Puerperal depression, developing around the third week after birth, was typified by Pitt as involving an increasing number of ‘bad’ days.

Depression is accompanied by anxiety, especially over the baby, and irritability, particularly towards the spouse and any other children, and tends to worsen as the day wears on.... Fatigue is extreme, and contributes to difficulty in coping... Despite the tiredness there is often great difficulty in getting to sleep.^46^

The interventions of Pitt and others, including studies based on general practice data, resulted in the wider adoption of the term puerperal and postnatal depression.^47^ Even so, its status, and that of puerperal psychosis, remained ambiguous; both, significantly, were accorded scant attention in the many editions of David Henderson’s influential textbook of psychiatry.^48^ In 1987, puerperal psychosis dropped out of the *DSM-III*, and even at the end of the century *The Shorter Oxford Textbook of Psychiatry* still explained that there is ‘no clear relationship between psychosis and obstetric factors’.^49^ Nonetheless, it was widely agreed in the clinical literature by the 1980s that while postpartum psychosis affected one or two mothers per 1,000, around one woman in 10—an estimate also given by Pitt for cases at the London Hospital in 1965—would experience postnatal depression.^50^

In practice, despite Pitt’s affirmation of clear categories and widespread acceptance of these rates of occurrence, definitions of postnatal depression remained hazy, and the number of women reported as experiencing depression in the weeks and months after delivery might, as shown below, vary significantly. While some practitioners continued to question the existence of a separate set of categories of mental disorder related to childbirth, other midwives, health visitors and doctors referred to a wide range of emotional disturbances affecting women after birth without labelling them as postnatal depression: anxiety, worry, stress, fear, confusion and despair. In 1979, for example, mid-wife Jean Ball conducted ‘an attitude survey’ exploring mothers’ emotional well-being after delivery, reporting that 25 per cent of her sample of 178 women were emotionally distressed, with many in this group complaining about the conflicting advice offered by midwives and being ‘too tired to care’.^51^ Yet, despite the shakiness of the boundaries between postnatal mental illness and other forms of emotional distress around child-birth, there is no doubt that the term postnatal depression had entered the mainstream by the early 1970s. This was evidenced by growing coverage in the press, women’s magazines and publications such as *Parents* and *Mother & Baby*, as well as NCT publications, and conversations about childbirth and postnatal depression also became more widely acceptable.^52^ As Fabiola Creed has demonstrated, the popular BBC radio programme, *Woman’s Hour* included numerous features on maternity and childbirth in the post-war years, and in 1960 aired its first feature on postnatal depression, an interview with psychiatrist Dr Russell Barton, followed by further features after the mid-1970s which involved women giving accounts of their own experiences.^53^

## Anti-Psychiatry, the Feminist Health Movement and the NCT

Increased interest in postnatal depression coincided with two important and overlapping movements: anti-psychiatry and the feminist health movement. By the 1970s, psychiatry was provoking ferocious criticism amongst the women’s movement in Britain, and doubts were cast on the specific labels used to describe women’s psychiatric illnesses. Aside from incarceration and inhumane treatments, including ECT and drug therapies, ‘feminists attacked diagnoses of mental illness for ignoring the fundamental social causes of widespread female unhappiness’.^54^ Mathew Thomson has described how by the early 1970s the women’s movement was ‘actively fashioning its own psychological positions and its own therapeutics’, and Kate Mahoney has shown how the Women’s Liberation Movement (WLM) sought to apply psychological and psychotherapeutic approaches to understand themselves politically and personally and to develop community-based support for women experiencing mental health concerns.^55^ Meanwhile, Sarah Crook has highlighted how the WLM developed an infrastructure (magazines, campaign and caring organisations, and consciousness-raising groups) through which ideas about ‘maternal distress’ more broadly could be communicated and disseminated, adopting much of the medical terminology, notably postnatal depression, while advocating social solutions.^56^

While questioning and criticising increasingly technological approaches in obstetrics, the NCT appears to have engaged far less with critiques of psychiatry, maintaining that for some women medical interventions, including drug therapies and ECT, might be beneficial. Nor did the NCT resist engagement with John Bowlby’s attachment theory, which had provoked the ire of the feminist movement, given its focus on centring the child’s welfare and development on the mother-child bond, ‘and its claim that separation of mothers from young children resulted in psychological deprivation’.^57^ This might be explained by the close association of the NCT with psychiatrists who, while highly critical of the medicalisation of obstetrics for its impact on mothers’ mental health, appear not to have questioned their own use of medical therapies. One of the NCT’s key supporters, Dr Desmond Bardon, was an early advocate of specialist psychiatric Mother and Baby Units, which aimed to prevent maternal deprivation by admitting mothers and babies together. While Bardon, as discussed below, eschewed medical approaches to childbirth, at the Unit he ran at Shenley Hospital, alongside psychotherapy, anti-depressants and tranquillisers were widely prescribed, and ECT used in a small number of cases.^58^

Far from considering postnatal mental illness solely as a medical phenomenon, however, the NCT strongly asserted the importance of sociocultural causes. In its publications and packs on postnatal depression, the NCT pointed to a range or combination of medical, psychological, social and cultural factors that might be responsible. Current practices in obstetrics resulting in poor experiences of childbirth were regarded as key, but NCT literature also referred to the vulnerability of women who had experienced previous mental illness and a range of social issues. These included poverty and poor housing, changes in women’s lives following on from motherhood that might leave them frustrated, over-worked or isolated, loss of status and identity when women gave up work to care for young children, social isolation and the stigma related to lone motherhood, moving away from family and other support networks, and exhaustion resulting from sleepless nights and the care of several young children. In this sense the NCT appeared to have traversed ‘the two bodies of work that have been most influential in this area of research as well as in popular beliefs about postnatal depression... the medical model and theories put forward by feminist social scientists’.^59^ Natasha S. Mauthner has defined the key features of these approaches, with the medical model focusing on the pathological condition rooted in deficiencies in the individual mother as the locus of explanation, while feminist theories emphasised the sociopolitical context, women’s experiences in relation to their circumstances, the people around them and the cultural and social conditions in which mothering occurs.^60^ In terms of practical responses to postnatal illness, to be discussed in the next section, the NCT also highlighted both medical and social causes and solutions.

Alongside its castigation of psychiatry and psychology, the feminist health movement criticised the medicalisation of childbirth and particularly the ‘reign of technology’ with regard to antenatal care and childbirth that Ann Oakley has described as ‘a strategy for the social control of women’.^61^ In 1980, Oakley explored postnatal depression in detail in *Women Confined*, suggesting that it was in particular de-personalised, medicalised and unsympathetic obstetric treatment that resulted in depression.^62^ Ellen G., who was given an epidural and had a manual removal of the placenta under general anaesthetic, described her birth as ‘a nightmare’, and the woman who administered the epidural as ‘a right... bastard’. Maureen P. also blamed her miserable hospital experiences and forceps delivery under general anaesthetic for her subsequent depression.^63^ Sharing Oakley’s opinion concerning the damaging impact of technological approaches in childbirth, along with isolation, overwork and segregated marital roles, the NCT’s information pamphlet on postnatal depression published a few years later in 1983, directly cited Oakley’s findings:

If women’s own feelings about labour are added to the analysis, it is clear that not enjoying and not experiencing achievement in labour constitute a further deprivation that, cumulatively with high technology and social vulnerability, provides a hazardous start to motherhood.^64^

This was confirmed by the accounts of NCT mothers included in the pamphlet, who attributed their depression to poor birth experiences. Rachel, for example, explained how her traumatic hospital birth, involving induction, a forceps delivery and ‘a lot of stitches’, made her ‘desperate, despairing, terrified, doomed, confused, angry’.^65^

As Lynn Abrams has suggested, the NCT had its roots in a self-help approach that was shared by other grassroots women’s organisations in the decades following the Second World War, which gave women experience of organisational work, lobbying and building support networks, as well as ‘retaining or reframing a sense of self’ as they became mothers.^66^ By the 1970s, the NCT was more closely aligned with feminist critiques of medicine and interventionist childbirth (evidenced in their adoption of several of Oakley’s publications on their reading lists) and advocacy of women’s right to take control of their own reproductive and birth experiences. Yet the organisation’s relationship with feminist health movements remained complex and poorly defined. Vivienne Welburn, a playwright and journalist who published one of the first personal accounts of postnatal depression in 1980, suggested that the NCT was engaged in ‘feminist activism’, ‘in that it gives women the knowledge which enables them to feel in control of their bodies’.^67^ Yet, she concluded, it remained positioned somewhat awkwardly between medicine and feminism, having been ‘attacked by conservative doctors and radical feminists alike. The doctors see it as radical and the feminists see it as conservative.’^68^ Welburn herself, in advocating social and practical solutions to postnatal mental illness—including help with childcare and the opportunity for women to share their problems—straddled the views of the feminist movement and the NCT. Her book was responded to positively in feminist publications; a letter to *Spare Rib* in 1981, for example, strongly recommended the book for its common-sense approach and questioning of medical professionals.^69^ In turn, Welburn’s book, which also featured prominently on NCT reading lists, warmly recommended the postnatal support offered by the NCT, particularly the use of experienced lay councillors to help depressed mothers, and Welburn was a regular speaker at NCT study days on postnatal mental illness.^70^

In practice, there was much engagement with the NCT in feminist publications. The British edition of *Our Bodies, Ourselves*, first published in 1978, urged mothers experiencing depression to contact the NCT or a self-help group such as the Association for Postnatal Mental Illness or MAMA (Meet-a-Mum Association) for support, and included an extensive list of NCT publications in its resources section.^71^ In May 1982, an article in *Spare Rib* listed the variety of causes put forward by what were framed as ‘experts’ to explain postnatal depression. These included hormonal changes, psychological theories proposing that it was a result of a woman’s refusal to adjust to her femininity and the role of mother, and problems such as poor housing and marital disharmony, a range of explanations recognisable to NCT members from the organisation’s own resource packs and publications. The impact of practices in maternity wards during labour was particularly highlighted in the article, citing the findings of a survey conducted with the NCT which related depression following childbirth to a loss of control during the delivery. According to the article in *Spare Rib*, a remarkable 74 per cent of women who said control had been taken by others stated that they felt depressed in hospital after the delivery. Technological interventions, including enemas, episiotomies, induction and epidurals, all led to higher scores on the scale measuring depression.^72^

## NCT Support for Postnatal Mental Illness

While careful to point to further sources of advice and assistance, including other charities and self-help groups, health visitors, midwives and GPs (though also casting doubt on the quality and availability of some of these resources), the NCT carved out an important role in offering information and support to women experiencing postnatal mental illness. With a network of area organisers, teachers, supporters and members, and tethered to the organisation’s long-term promotion of emotional alongside physical wellbeing in child-birth, the NCT was in a strong position to develop this kind of work. Focusing principally on postnatal depression as the condition most likely to confront their membership, the NCT emphasised in their antenatal classes the need for women to be prepared for feelings of depression and anxiety after delivery. After the late 1960s, the NCT focused increasingly on postnatal support, and by the early 1980s, the organisation was producing resource packs, pamphlets and publications on caesarean section, miscarriage, stillbirth and bereavement, breastfeeding, crying babies, babies in special care, parentability (advice to disabled parents), the role of fathers and postnatal depression. Postnatal classes and networks were set up, which often involved women who had themselves experienced postnatal depression.

The NCT highlighted support, contact with other mothers and friendship above all else as key to helping women overcome postnatal depression, as a vital accompaniment to medical care, and as something that the NCT was well equipped to offer. In a pamphlet published by the NCT in 1974 outlining some of the challenges of the first weeks of motherhood, Margaret Dennis advised new mothers swamped with information on postnatal depression not to ‘expect to experience it as a natural sequel to having a baby’, and emphasised its curability and the help offered by doctors.^73^ Dennis had built up extensive counselling experience, particularly with mothers suffering postnatal depression, was an active member of the Oxford Post-Natal Support Group, and had experienced depression herself after the birth of her second child.^74^ Though positive about the likelihood of recovery, she warned mothers who were feeling ‘down’ or exhausted that they might slide into postnatal depression, and urged those experiencing early signs to seek out a NCT support group.^75^ Community midwife, health visitor and NCT teacher Antoinette Ward was involved in supporting women experiencing postnatal depression during the early 1980s, and in a short memo ‘Knowing my Limitations’ set out a lengthy and diverse list of potential causes as she saw them. These included medical, social and emotional factors: latent mental illness and hormonal imbalance, financial worries, poor housing, difficult marital relationships or relatives, the loss of a job and status following childbirth, and isolation and fatigue, resulting from a lack of sleep, night feeding and a restless crying baby. Ward also referred to the vulnerability of women of an ‘immature personality with a consequent inability to cope’, reflecting a more commonly held view in social work that some mothers were failing due to their own inadequacies.^76^ Her advice for helping women who were depressed included listening attentively and non-critically, and questioning constructively, offering practical help, trying to find out how women explained their depression, encouraging solutions and ideas about recovery, and communication between the woman and her husband, family and friends. Ward also pointed out that medical help, including hospitalisation and medication, might be necessary.^77^

I’m not trying here to suggest that we should all try to be amateur psychiatrists—I feel that all the points I have made arise out of common sense and the skills involved are those we use in friendship which is what we are really offering. We may well feel that a woman needs medical help, perhaps drugs, hospitalisation, group therapy or ECT but she also needs the mother-to-mother support and encouragement to be found in her local NCT branch or group. So let me both know my limitations and at the same time recognise my abilities.^78^

Ward’s approach was reflected in much of the literature produced by the NCT, as information on postnatal depression evolved into separate section of a pack on postnatal support, and by 1987 into a 46-page resource pack devoted solely to postnatal depression. Again, this material referred to the range of medical, social and emotional causes that might prompt depression. It stressed the need to de-stigmatise postnatal mental illness and to have open conversations about its impact, and emphasised the importance of offering a ‘listening ear’ but not giving advice. They are there to support the woman, the pack explained, ‘not to advise, diagnose, counsel or judge’.^79^ The NCT acknowledged the fact that supporters too might need back up, and that supporting someone with postnatal depression might be emotionally and physically tiring and need to be shared.^80^ NCT support networks were also seen as having the potential to enable mothers to take the first step in seeking help.

Unlike a broken limb, postnatal depression is unseen and women often struggle on alone resisting the need to ask for help. They may be unable to admit that help is needed. There is the added problem of the illness being misunderstood and regarded in some circles as a taboo subject. Women may feel isolated, guilty and a failure at a time when they feel they should be happy and enjoying the experience of being a parent.^81^

The need to be sensitive to issues raised when the women supported were of different religious faiths, cultures or backgrounds was also pointed out. In these cases, it was suggested, great stigma might be attached to psychiatric illness and the barriers to seeking help or revealing symptoms of postnatal depression might also be greater. These comments appeared to be acknowledging criticisms levelled at the NCT for focusing largely on white, middle-class women.^82^ While some women, it was claimed, might recover when offered practical and emotional support, it was also suggested that supporters should encourage women to seek medical help if the condition persisted or became worse. A supporter must never recommend drugs or other forms of treatment, but their role might be to encourage women to keep taking prescribed medication, and the pack also pointed out that counselling or complementary therapies, hormonal treatments or ECT might be necessary.^83^ Supporters were urged to act on the side of caution and to go further in intervening if ‘you feel there is a risk, no matter how small, of suicide or infanticide’, by encouraging the woman to contact a doctor or health visitor, to do this together or, as a last resort, to take action on her behalf with or without permission.^84^

Increasingly, the NCT included articles in its publications on the theme of postnatal mental illness, and after 1968 its newly launched magazine, *New Generation*, included contributions on depression after childbirth written by health professionals as well as personal recollections.^85^ The magazine also contained numerous reports on the growing number of study days, workshops and conferences devoted to postnatal mental illness, which were intended to be a ‘neutral meeting ground’ involving lay people and ‘consumers’ as well as medical professionals.^86^ In June 1980, Dr Katharina Dalton was the main speaker for a Study Day on Post-Natal Depression held in Doncaster, along with a panel made up of a consultant psychiatrist at the local infirmary, a senior clinical psychologist, and a mental health case worker. While NCT postnatal supporters were present at the meeting, the focus—according to the press report—was less than neutral and emphasised medical solutions, including anti-depressants and the hormonal therapy that Dalton was spearheading. Practical advice, however, was also given. Dalton urged family and friends not to nag the ‘lethargic and unkempt mother’ about a sink full of dishes or untidy house. ‘Don’t say “Pull yourself together”‘.^87^ In June 1985, a report on a symposium on postnatal depression held in Glasgow regretted the dominance of professional voices and the small number of mothers attending, and referred to an ‘intolerable burden of scepticism’ about postnatal mental illness.^88^ During a film showing which related to the ‘depressed mums themselves’ and compared ‘symptoms revealing suicidal tendencies, talking of deep and terrible feelings and emotions’, many of the professionals were reported to be ‘bored and fidgety, they wanted to know facts about treatment, cure, research, etc.’^89^ At other events, however, women centred the discussion around their concerns and viewpoints. In November 1981, a Postnatal Depression Workshop for postnatal supporters and coordinators was held at NCT headquarters. It featured a talk by Vivienne Welburn describing her experiences of depression, ascribed to a failure to meet her own expectations of motherhood and a lack of help with her second baby. The day focused largely on experiences of birth and social issues, identifying causes of depression in the birth experience itself and stressing the importance of mothers giving birth in ‘the way which is most important for *her*’, the over-idealisation of motherhood, the sense of loss associated with a change in identity, social isolation and physical exhaustion.^90^

## Expertise, Women’s Voices and the NCT

The fact that the NCT highlighted a broad range of medical, psychiatric, emotional and socioeconomic causes of postnatal mental illness provided scope for engagement with many experts with an equally diverse range of viewpoints. Sheila Kitzinger, one of the NCT’s most influential supporters, evolved her own approach to natural childbirth, emphasising social and psychological preparedness above technique and exercise. Preparation for natural birth, in her opinion, would reduce the likelihood of ‘extremes of emotional unpredictability’ and lead to a fulfilled birth experience. Analysing the responses of women attending NCT classes about the place and their experiences of delivery, and their preference for home or hospital confinements, Kitzinger showed that the closeness of mother and baby in home deliveries was an advantage for bonding and a means of avoiding depression, as home births were associated with a more restful postpartum experience.^91^ In contrast, she explained that she saw several new cases each week of emotional problems associated with hospital birth. Mrs Y, suffering from postnatal depression nine months after the birth of her second child, who had been delivered by forceps and then quickly removed from the mother, described how after the delivery ‘I was in limbo. It was an unreal feeling. Had I had the baby or hadn’t I?’^92^ Kitzinger pointed out that one in 10 women received medical treatment for depression after birth. This was, in her view, ‘the tip of the iceberg’. Depression, Kitzinger argued, was less to do with hormonal disturbance, and was more likely caused by feeling unable to conform to the expectations of new motherhood, socioeconomic stress or a disempowered hospital birth given ‘the institutional violence typical of care in many hospitals’.^93^

Sheila Kitzinger’s powerful argument that home birth, choice and agency were likely to reduce the incidence of emotional stress and depression resonated with the NCT’s broader objectives. Yet, the organisation also worked closely with other acknowledged experts in the field, who took a more medical approach. Katharina Dalton’s *Depression after Childbirth*, which explained that hormonal change caused postnatal depression and hormonal treatments could cure it, was highlighted by the NCT as a useful resource, and Dalton was an active supporter of the NCT’s work in this area.^94^ While it was pointed out by the NCT that not everyone agreed with Dalton’s premise that postnatal depression was caused by hormonal imbalance and required hormonal treatment, she was credited with a warm and sympathetic approach and offered hope of prevention and cure.^95^ The NCT’s eclectic approach was also reflected in its reading lists on postnatal depression. Alongside, Oakley’s feminist analysis in *Women Confined*, popular psychiatric texts, such as John Cobb’s *Babyshock*, were listed, though Cobb questioned whether bad experiences during labour, including drugs, induction and caesarean section, could ‘have *on their own* been responsible for prolonged emotional upset’.^96^ An expanding range of books based on personal experiences were also recommended.^97^

Psychiatrists specialising in postnatal mental illness, particularly those working with the NCT, were largely critical of obstetric practices, emphasising poor experiences of birth, loneliness and isolation, and suggesting that the hospital itself as a place of birth provoked mental breakdown. In 1984 respected Edinburgh psychiatrist R.E. Kendell, referred to a stepped up interest in psychiatric disorders associated with childbirth over the past decade, which coincided with concern for ‘the emotional hazards of childbirth... as obstetric units become increasingly dominated by gadgetry’.^98^ Desmond Bardon, psychiatric advisor to the NCT during the 1970s and 80s, and admirer of Norman Morris, corresponded regularly with its officers on such matters, enclosing copies of his articles and conference papers. Though ambivalent about the uniqueness of puerperal depression compared with other depressive disorders, Bardon used the term repeatedly in his publications and conference papers, which were largely directed towards educating general practitioners, midwives and health visitors. He argued that the hospital failed to meet the emotional needs of mothers, their poor treatment during delivery by doctors and nurses often leading to ‘depressing humiliation, remembered with disappointment and resentment’.^99^ He also criticised staff shortages, inadequate communication, the lack of advice on baby care and breastfeeding, as well as particular procedures, such as the use of anaesthesia or forceps, which damaged women’s self-esteem and disturbed attachment processes.^100^ ‘There is little doubt’, he claimed, ‘that the incidence of post puerperal depression, of emotional disorder generally, and of disturbance in the mother-baby relationship, are less frequent in home confinements than in hospital confinements.’^101^

While complaining about an absence of statistical evidence, Bardon cited the research of Joan Court, midwife, social worker and ‘battered baby’ expert, who concluded that the ‘depressive reaction’ was not observed in mothers who gave birth at home or in institutions where mother and baby were kept together.^102^ A.A. Baker, consultant psychiatrist at Banstead Hospital and the Mother and Baby Unit at Downview Hospital, whose work was also cited by Bardon, observed that ‘major psychoses develop very much more frequently in mothers delivered in a maternity unit than in mothers delivered at home’.^103^ Bardon provided figures taken from the research of Tod and Ryle showing rates of around 3 per cent in their general practices, while at Cardiff’s Department of Obstetrics and Gynaecology out of a sample of 193 women giving birth in hospital, 107 or 64 per cent became depressed compared with 19 per cent of the 86 delivered at home.^104^ The sample was small but the results striking, with figures greatly in excess of accepted rates of postnatal depression. Interpreting such findings is challenging and might indicate the limits of the sample size or a lack of rigour in data collection. They also suggest an eagerness to be inclusive (even overly inclusive) in terms of reporting signs of mental illness which highlighted poor results in hospital settings. Reinforcing the link between highly managed hospital births and postnatal mental illness, such figures might also have been drawn on by the NCT to amplify their concerns about the failures of technological childbirth, offering evidence to support the organisation’s campaigns for home births and the extension of women’s choice and agency.

At the same time as decrying the impact of hospital deliveries on new mothers and the risks this imposed on the mother’s mental health, Bardon, a devotee of Bowlby’s attachment theory, was at pains to point out that the mother’s depression was likely to have a detrimental impact on the infant’s wellbeing and development and family life more broadly. Stressing the importance of the first days of life to the newborn, in a letter sent in 1981 to ‘Ann’ [Bowen Jones, National Secretary to the NCT] Bardon described how separation was a particular risk for mothers predisposed to attachment failure, who had experienced emotional deprivation themselves in childhood, lacked confidence and had an impaired capacity for giving and receiving love.^105^ He argued that the most important stress factor in puerperal depression was psychological, ‘and that it arises from unconscious conflicts in the woman about measuring the role and responsibilities of mother’.^106^ Bardon cited particular examples of cases involving ‘unconscious conflicts in the woman about assuming the role and responsibilities of mother’, including ‘a depressed, aggressive mother who, when her 4 month old daughter cried, saw her as a sophisticated blackmailer’. The mother admitted being jealous of the child: ‘She is projecting her own unmet dependency needs and her continuing search for attention and affection onto her child.’^107^

While the challenges of getting their voices heard at some workshops and study days have been referred to above, the NCT was eager to draw on women’s own accounts of postnatal mental illness to present information to members and other mothers, regarding these women as another set of experts. Unlike those of Jennifer Crane’s ‘experts by experience’, these interventions might not have directly changed policy or practice. However, it is likely that they contributed towards breaking down stigma and opened up conversations about postnatal depression within and beyond the NCT. Most also offered hope of recovery.^108^ The importance of including women’s experiences had been highlighted in *New Generation* as early as 1968, when one woman explained how the strains of caring for a young family had triggered her depression, resulting in her feeling dreary, irritable and tired. She also described her recovery and saw her own depression as being ‘mild’, and attributable to her isolation on a new ‘industrial’ housing estate where depression was very common.^109^

Liz Waumsley, Chair of the NCT’s Postnatal Committee, highlighted the increased self-knowledge and strength shown by women writing accounts of their experiences in her foreword to the NCT’s 1983 booklet on postnatal depression.^110^ The booklet was largely devoted to women’s voices, offering insights into experiences of postnatal depression and recovery, albeit for some women after many months or even years.^111^ These accounts—unedited and often harrowing—described a range of issues that the women themselves identified as the root causes of their depression, including previous mental illness, being overburdened by the demands of the household and the needs of an infant, difficulties breastfeeding, and the isolation of being alone at home all day with a new baby or several young children. Like Rachel cited above, several described difficult birth experiences. Carol summed up the shock and disappointment of having a caesarean section, followed by ‘intense fatigue’, after a happy pregnancy and anticipation of a natural delivery.^112^ Margaret, who had experienced a 40-hour labour and painful experiences of breastfeeding, concluded that postnatal depression should not just be treated on a medical basis, though she took ‘tablets’ for six months. Echoing the comments of Dalton, she got ‘sick of being told “Pull yourself together”. That’s just what you can’t do.’ She also explained the need to speak to someone who had been through the same thing.^113^ Some of the women, however, barely referred to their birth experiences or were positive about them. Ann had ‘a good confinement in a homely GP unit’ and attributed her depression to difficulties breastfeeding and a loss of confidence, exacerbated by a house move to a new area shortly after the birth.^114^ It is also possible that the feelings of disappointment described by Carol were attributable to NCT information and classes that raised expectations about the benefits of natural birth. The women had mixed feelings about professional intervention, and several were critical of their GPs’ lack of knowledge and understanding, Hilary describing the comments of her GP as ‘positively hostile and often inhuman’.^115^ Others praised the support of doctors, health visitors and the NCT. Diane became closely involved in offering postnatal support in the NCT ‘so that had [*sic*] an interesting and useful “job” to do outside the home, and a lot of understanding friends’.^116^

*New Generation* continued to welcome the accounts of members who had experienced postnatal depression. A summary of a meeting at the Reading, Henley and District Branch, published in June 1982, captured the experiences of three women who had suffered postnatal depression, one of whom was training to be an antenatal teacher. All three related their depression to an unsatisfactory delivery and all ‘had high expectations of the birth experience that were not met’. Links were also made with traumatic childhoods, the need to be looked after, and the feeling that they weren’t coping. ‘Recognising the condition was crucial to [the] recovery of all of us’.^117^ Two of the women claimed that they had very high standards and were keen to do a better job of mothering than their own mothers, and all felt lonely. ‘A crucial question, of course, is the **effect our depression has on our children**, especially when we punish them. It’s hard to tell—certainly my daughter has been quite insecure and desperate for mummy’s approval at times. As I have recovered, our relationship has steadily improved.’^118^

These concerns, the difficulties felt in adapting to motherhood and feelings of estrangement and even dislike or jealousy towards their newborn child, reflected similar concerns to those raised by Bardon, and were echoed more widely. Rachel described herself as ‘a difficult mother’, who threatened ‘to do dreadful things to Alex’. Three years on and after a second child, she and Alex were much happier, but he was ‘still a bit of a problem... He is very clingy and insecure and has a tendency toward temper tantrums. But who can blame him after his bad start?’^119^ Many referred to a sense of failure as mothers, Margaret recalling ‘I felt totally inadequate as a mother... But at the same time I felt so totally responsible for James that I was frightened to leave him even for a couple of hours.’^120^ This raises the question of how far the women describing their anxieties about the impact of their depression on their children were prompted by their personal experience or steered by a broader knowledge of bonding and attachment theory that so permeated childcare literature and the media during this period. Donald Winnicott, who, through his books and radio programmes, influenced child development well into the post-war period, claimed that while no one would ‘blame’ the mother for suffering depression after giving birth, ‘she can feel herself depriving her child of what the child needs’.^121^ Working closely with experts, like Bardon, whose lengthy involvement with the NCT involved correspondence with NCT members and branches, and frequent contributions to its publications, might have had unintended consequences for the NCT, causing more anxiety for mothers experiencing postnatal depression. In an article published in *New Generation* in 1983, while praising NCT postnatal supporters, he advised the organisation to strive to take the ‘luck’ out of childbirth by ensuring that pregnant women know the conditions that could both ‘blight the birth and growth of love’.^122^

Given the organisation’s middle-class bias and focus on natural childbirth, some mothers felt excluded or that they were doing things wrong. Angela Davis has described how the NCT was often picked out by the women she interviewed in Oxfordshire about their experiences of motherhood as a way of meeting other mothers, and they emphasised the importance of what often evolved into informal arrangements and friendships. Shirley, for example, described the support of other mothers when she developed depression after the birth of her first child as crucial.^123^ Others, however, found the NCT meetings useless, as they prompted feelings of inadequacy, and some commented that they were dominated by middle-class women.^124^ Critical assessments of the work of the NCT featured even in its own publications. One new mother described how she ‘failed miserably as an NCT mum’, rejecting squatting to give birth and being unable to manage without drugs; her NCT teacher was described as ‘condescending’.^125^ Several of Davis’s Oxford based interviewees attended Sheila Kitzinger’s classes, and not all were impressed. Monika described how ‘Sheila Kitzinger was carrying on about this, and natural childbirth was a great cry. I wasn’t at all sold on natural childbirth. I thought the easier the better thank you very much.’^126^ Several also reported feeling under pressure to have as little medical intervention as possible, having ‘failed’ to meet the NCT’s high expectations, or that they had let Kitzinger down by having to have a caesarean section.^127^

## Conclusion

Monika’s comment above is telling. With the push to encourage natural childbirth and home births and to emphasise the disadvantages of the hospital and technological interventions, the NCT and its supporters might well have missed the point that some women would not be attracted by the idea of choice and may have preferred a managed hospital delivery. It also highlights the enormous complexities surrounding the issue of choice in childbirth that women were faced with in the post-war period, and the push and pull of those advocating natural childbirth on the one hand and managed hospital births on the other. Exploring the NCT and postnatal depression also reveals fissures within professional viewpoints and practices, with some doctors providing support for NCT campaigns urging natural childbirth and women’s agency and pointing to an additional risk factor of technological births—the mother’s mental breakdown—while others continued to champion the push towards medicalised births. While resisting medicalisation and asserting the rights and choices of its consumer mothers aligned with feminist objectives, the NCT had an ambiguous relationship with the feminist health movement, and, while having a clear line on obstetric intervention, offered few challenges to psychiatry and its therapeutic approaches or the pressures placed on mothers by attachment theory.

While the NCT gave voice to a range of expert opinion, and at the same time promoted women’s choice and agency, the organisation’s advocacy of natural birth and criticism of the move to interventionalist hospital birth was widely shared amongst these experts. Thus, women were given a strong steer concerning their choices, which were also likely to have raised expectations of what a natural birth should be like. This might have produced feelings of failure, and potentially symptoms of depression, amongst women unable to achieve the NCT’s idealised good birth. The NCT also drew on the connection between managed hospital births and poor mental health outcomes to highlight concerns about the increased use of childbirth technologies. That said, there is no doubt that many women came to the same conclusion, blaming their depression on dreadful experiences of hospital deliveries and excessive and often unanticipated intervention. It is also possible that by giving voice to concerns about the impact of postnatal mental illness on bonding and their newborns’ development, the NCT exacerbated the misery of postnatal depression for some mothers. While Angela Davis has cautioned against the idea that mothers automatically accepted and responded to expert advice on childbirth and motherhood, clearly some mothers either took on board the core ideas of attachment theory or developed similar ideas regarding the impact of their illness based on their own experiences.^128^

There were also persistent challenges for the NCT. While some members complained about the unevenness of the postnatal support on offer, which was described by some as non-existent or unsatisfactory, by the late 1980s, the organisation itself was expressing concern about its limited reach.^129^ Though it is notable that a new NCT pamphlet on postnatal depression, published in 1990, featured a black mother and her baby on its front cover, when data was collected on membership in the same year, figures on race and ethnicity were not included, and most members were still recorded as middle class.^130^ Notwithstanding limitations in terms of their reach, there is little doubt, however, that the NCT contributed to efforts to destigmatise postnatal mental illness and opened up discussions on the subject, which engaged supporters, mothers and health professionals. The organisation gave mothers a voice in describing their own experiences of postnatal depression and enabled them to build up their knowledge and exchange information at workshops and study days, even if, as shown above, their voices might on occasion be muted. The NCT also exposed mothers to a multitude of different viewpoints on causality and treatment. Though emphasising the impact of poor birth experiences, the organisation drew attention to many other factors that might prompt postnatal depression, emotional, social and medical. Through its classes and support networks, the NCT sought to develop a sense of community amongst mothers, emphasising the benefits of women supporting each other, which also addressed the issue of shortfalls in professional help and in professional knowledge and understanding of postnatal mental illness. As Bardon concluded, women’s support was necessary. ‘There aren’t enough professionals to go round. There never will be. Health is only marginally in the gift of doctors. All societies, in a sense, must will and create their own health.’^131^ One could be sceptical about the impact that an approach based largely on support might have on women experiencing postnatal depression. However, loneliness and isolation was, and continues to be, emphasised by health professionals and women who experience postnatal mental illness as likely to make mothers vulnerable to depression, while support, particularly peer support, is recognised as an essential component of recovery.^132^

## Footnotes

1 Jane Lewis, ‘Mothers and Maternity Policies in the Twentieth Century’, in Jo Garcia, Robert Kilpatrick and Martin Richards, eds, *The Politics of Maternity Care: Services for Childbearing Women in Twentieth-Century Britain* (Oxford: Clarendon, 1990), 15–29; Caitriona Beaumont, *Housewives and Citizens: Domesticity and the Women’s Movement in England, 1928–64* (Manchester: Manchester University Press, 2013), ch. 4; Hilary Marland, ‘Childbirth and Maternity’, in Roger Cooter and John Pickstone, eds, *Medicine in the 20*th *Century* (Amsterdam: Harwood, 2000), 559–74. For the National Birthday Trust Fund, see Wellcome Collection (WC), SA/NBT/G.5/1, An Account of the Work carried out by the National Birthday Trust Fund, November 1947; Susan A. Williams, *Women and Childbirth in the Twentieth Century: A History of the National Birthday Trust Fund 1928–93* (Stroud: Sutton Publishing, 1997).

2 Irvine Loudon, *Death in Childbirth: An International Study of Maternity Care and Maternal Mortality 1800–1950* (Oxford: Oxford University Press, 1992); Elizabeth Peretz, ‘A Maternity Service for England and Wales: Local Authority Maternity Care in the Inter-War Period in Oxfordshire and Tottenham’, in Garcia, Kilpatrick and Richards, eds, *The Politics of Maternity Care*, 30–46; Angela Davis, ‘A Revolution in Maternity Care? Women and the Maternity Services in Oxfordshire c.1948–1974’, *Social History of Medicine*, 2001, 24, 389–406; Beaumont, *Housewives and Citizens*, ch. 4.

3 Angela Davis, *Modern Motherhood: Women and Family in England, 1945–2000* (Manchester: Manchester University Press, 2012), 84; see also Tania McIntosh, *A Social History of Maternity Care* (Abingdon: Routledge, 2012).

4 For example, see Helen McCarthy, *Double Lives: A History of Working Motherhood* (London: Bloomsbury, 2020); Martin Pugh, *Women and the Women’s Movement in Britain, 1914–1999*, 3rd edn (London: Palgrave Macmillan, 2015); Ina Zweiniger-Bargielowska, ed., *Women in Twentieth-Century Britain* (Harlow: Pearson, 2001); Hera Cook, *The Long Sexual Revolution: English Women, Sex, and Contraception 1800–1975* (Oxford: Oxford University Press, 2005).

5 For example, see Lesley Doyle, ‘Women, Health and the Sexual Division of Labor: A Case Study of the Women’s Health Movement in Britain’, *International Journal of Health Services*, 1983, 13, 373–87; Zoe Strimpel, ‘*Spare Rib*, the British Women’s Health Movement and the Empowerment of Misery’, *Social History of Medicine*, 2022, 35, 217–36.

6 See R. Campbell and A. Macfarlane, *Where to be Born? The Debate and the Evidence* (Oxford: National Perinatal Epidemiology Unit, 1987); Marjorie Tew, *Safer Childbirth? A Critical History of Maternity Care* (London: Chapman and Hall, 1990).

7 See, for policy in maternity services, Angela Davis, ‘Choice, Policy and Practice in Maternity Care since 1948’, *History & Policy*, 2013: https://www.history-andpolicy.org/policy-papers/papers/choice-policy-and-practice-in-maternity-care-since-1948; McIntosh, *A Social History of Maternity Care*.

8 WC, PP/GDR/F12, Dick-Read, Grantly, 1890–1959, National Childbirth Trust, 1957–69, Some Thoughts on Press Interviews for AOs [Area Organisers] and Helpers (March 1962); Jenny Kitzinger, ‘Strategies of the Early Childbirth Movement: A Case-Study of the National Childbirth Trust’, in Garcia, Kilpatrick and Richards, eds, *The Politics of Maternity Care*, 92–115, 93; https://www.nct.org.uk/about-us/history.

9 See Page L., ‘Kitzinger [nee Webster], Sheila Helena Elizabeth (1929–2015)’, *Oxford Dictionary of National Biography* (*DNB*) (accessed 3 Aug. 2022): https://www.oxforddnb.com.pugwash.lib.warwick.ac.uk/view/10.1093/odnb/9780198614128.001.0001/odnb-9780198614128-e-110338

10 McIntosh, *A Social History of Maternity Care*, 114.

11 WC, SA/NCT/H/1/2/3, Grantly Dick-Read Centenary and Papers Relating to History of NCT, 1956–90, Letter from Prunella Briance to Editor of *New Generation*, 1986. See, for responses to Dick-Read’s work, Mary Thomas, ed., *Postwar Mothers: Childbirth Letters to Grantly Dick-Read 1946–1956* (Rochester, NY: University of Rochester Press, 1997).

12 Grantly Dick-Read, *Introduction to Motherhood* (New York: Harper & Brothers, 1950), 33–36.

13 Grantly Dick-Read, *Natural Childbirth* (London: Heinemann, 1933).

14 Grantly Dick-Read, *Motherhood in the Postwar World* (London: Heinemann, 1944); Ornella Moscucci, ‘Holistic Obstetrics: The Origins of “Natural Childbirth” in Britain’, *Postgraduate Medical Journal*, 2003, 79, 168–73, 169, 171; WC, PP/GDR/1/F12, Dick-Read, Grantly, 1890–1959, National Childbirth Trust, 1957–69, NCT Newsletter, No. 2 (March 1957).

15 Williams, *Women and Childbirth*, 143, 201.

16 WC, SA/NCT/H/1/2/3, Letter from Prunella Briance to Editor of *New Generation*, 23 March 1986.

17 Erna Wright, *The New Childbirth* (London: Tandem, 1964). For Lamaze, see Paula A. Michaels, *Lamaze: An International History* (New York: Oxford University Press, 2014).

18 Paula A. Michaels, ‘The Sounds and Sights of Natural Childbirth: Films and Records in Antenatal Preparation Classes, 1950–1980s’, *Social History of Medicine*, 2017, 31, 24–40, 35, 29; Moscucci, ‘Holistic Obstetrics’, 172; WC, PP/GDR/1/F12, NCT Newsletter, No. 2 (March 1957).

19 WC, PP/GDR/1/F12, NCT Newsletter, No. 10 (July 1962); WC, SA/NCT/A/1/1/2, Landmarks/Documents of historical interest: early NCT correspondence and papers, 1952–66, NCT Annual General Meeting, November 1958, 2.

20 WC, SA/NCT/H/1/2/3, Typescript ‘The Founding of the National Childbirth Trust’, n.d.

21 WC, PP/GDR/1/F12, Mr. Perchard’s Notes on Relations of N.C.T. Members with Doctors and Midwives, n.d. [c.1962].

22 Ian Donald, *Practical Obstetric Problems*, 3rd edn (London: Lloyd-Luke (Medical Books), 1964), 444, 429.

23 Ibid., 444–45.

24 Norman Morris, ‘Human Relations in Obstetric Practice’, *The Lancet*, 23 April 1960, 913–15.

25 Ibid., 913.

26 Ibid., 914. Morris’s views were confirmed in press accounts, including a piece on ‘Callous Midwives’, *Sunday Dispatch*, 13 November 1960, describing women’s terrifying experiences of hospital deliveries.

27 Central Health Services Council. Standing Maternity and Midwifery Advisory Committee, ‘Human Relations in Obstetrics’ (London: HMSO, 1961), 2.

28 Joanna Moorhead, *New Generations: 40 Years of Birth in Britain* (Chesterton: HMSO in collaboration with National Childbirth Trust Publishing, 1996), 35.

29 Jean Soward, ‘Our Maternity Services’, *The Guardian*, 18 January 1961.

30 WC, SA/NCT/J/2/2/1, ‘Retiring Chairman’s Introduction’, *New Generation*, October 1967, 1:1, 5–7, 7.

31 Davis, *Modern Motherhood*, 87.

32 Ivan Illich, *Medical Nemesis* (London: Calder & Boyars, 1974).

33 WC, PP/DGR/F12, ‘Some Thoughts on Press Interviews’ (March 1962); Kitzinger, ‘Strategies of the Early Childbirth Movement’, 93; Marland, ‘Childbirth and Maternity’, 573. See Alex Mold, *Making the Patient-Consumer: Patient Organisations and Health Consumerism in Britain* (Manchester: Manchester University Press, 2015).

34 Sheila Kitzinger, *The Good Birth Guide* (London: Fontana, 1979).

35 Margaret Allen, ‘Viewpoint’, *The Times*, 13 August 1973, 8.

36 John Illman, ‘Birth of a Revolution: Mothers Win their Fight for Better Treatment’, *Daily Mail*, 12 October 1983, 12. Multiple examples of the promotion of home birth and improvements in maternity care are located in NCT press cuttings: WC, SA/NCT/M/1.

37 Michaels, ‘The Sounds and Sights of Natural Childbirth’.

38 Hilary Marland, *Dangerous Motherhood: Insanity and Childbirth in Victorian Britain* (Houndmills: Palgrave Macmillan, 2004), chs. 2, 7.

39 Jean Hwang, ‘Women and Depression in Interwar Britain: Casenotes, Narratives and Experiences’, unpublished PhD thesis, Warwick University’, 2018, 230–31, shows the declining but still persistent use of the term puerperal insanity in *The Lancet* and *Journal of Mental Science* up until the 1940s.

40 For example, D.N. Parfitt, ‘Psychoses Associated with Childbirth’, *Journal of Mental Science*, 1934, 80:328, 43–57. ‘Insanities of reproduction’ was more likely to be used in obstetric literature, such as Henry Jellett and David G. Madill, *A Manual of Midwifery for Students and Practitioners*, 4th edn (London: Baillière, Tindall & Cox, 1929), 1038–44.

41 See Marland, *Dangerous Motherhood*, ch. 7; Bethel Solomons, ‘Insanity and its Relation to the Parturient State’, *Journal of Mental Science*, 1931, 77:319, 701–07; Parfitt, ‘Psychoses Associated with Childbirth’.

42 Sarah Crook, ‘The Uses of Maternal Distress in British Society, c.1948–1979’, unpublished PhD thesis, Queen Mary University of London, 2016, 10.

43 A.B. Hegarty, ‘Post-Puerperal Recurrent Depression’, *British Medical Journal*, 12 March 1955, 637–40, 637.

44 Brice Pitt, ‘“Atypical” Depression Following Childbirth’, *British Journal of Psychiatry*, 1968, 114:516, 1325–35, 1325.

45 Ibid., 1325; Brice Pitt, *Psychiatric Problems in Women. Part 1: Puerperal Depression* (Welwyn Garden City: SK&F Publications, 1981).

46 Pitt, *Psychiatric Problems*, 6.

47 A. Ryle, ‘The Psychological Disturbances Associated with 345 Pregnancies in 137 Women’, *Journal of Mental Science*, 1961, 107:447, 279–86; E.D.M. Tod, ‘Puerperal Depression: A Prospective Epidemiological Study’, *The Lancet*, 12 December 1964, 1264–66.

48 For example, less than a page in *Henderson and Gillespie’s Textbook of Psychiatry for Students and Practitioners*, 10th edn (London, New York and Toronto: Oxford University Press, 1969).

49 Michael O’Hara, *Postpartum Depression: Causes and Consequences* (New York: Springer-Verlag, 1995), 3; *DSM-III, the 3rd edn of the Diagnostic and Statistical Manual of Mental Disorders from the American Psychiatric Association* (New York: American Psychiatric Association, 1980); Richard Mayou, Michael Gelder and Philip Cowen, *The Shorter Oxford Textbook of Psychiatry* (Oxford: Oxford University Press, 2001).

50 R.E. Kendell, ‘Emotional and Physical Factors in the Genesis of Puerperal Mental Disorders’, *Journal of Psychosomatic Research*, 1985, 29, 3–11, 3; Brice Pitt, ‘Puerperal Depression’ (‘Letters to the Editor’), *The Lancet*, 9 January 1965, 107.

51 Jean Ball, ‘Stress and the Postnatal Care of Women’, *Nursing Times*, 10 November 1982, 1904–07, 1906.

52 See NCT press cuttings: WC, SA/NCT/M/1.

53 Fabiola Creed, ‘The Modernisation of Postnatal Depression through *Woman’s Hour*: Changing Agency, Voices and Treatments in Britain, 1960–1985’, forthcoming in Heather Love, Jerika Sanderson, Karen Weingarten and Whitney Wood, eds, ‘Making Modern Maternity’, *Medical Humanities*, special issue 2024.

54 Mathew Thomson, *Psychological Subjects: Identity, Culture, and Health in Twentieth-Century Britain* (Oxford: Oxford University Press, 2006), 279–80. See also Juliet Mitchell, *Psychoanalysis and Feminism* (New York: Pantheon, 1974); Oisín Wall, *The British Anti-Psychiatrists: From Institutional Psychiatry to the Counter-Culture, 1960–1971* (New York: Routledge, 2019).

55 Thomson, *Psychological Subjects*, 279; Kate Mahoney, ‘“Finding our own solutions”: The Women’s Movement and Mental Health Activism in Late Twentieth-Century England’, unpublished PhD thesis, Warwick University, 2017. See also Kate Mahoney, *Feminist Mental Health Activism in England, c.1968–1995* (Manchester: Manchester University Press, 2023).

56 Crook, ‘The Uses of Maternal Distress’, 141, 184–85; Sarah Crook, *Postnatal Depression in Postwar Britain: Women, Motherhood, and Social Change* (Manchester: Manchester University Press, forthcoming 2024), ch. 4.

57 Thomson, *Psychological Subjects*, 279. See, for the impact of Bowlby and his emphasis on home and family, Mathew Thomson, *Lost Freedom: The Landscape of the Child and the British Post-War Settlement* (Oxford: Oxford University Press, 2013), ch. 3, and for the popularisation of Bowlby’s theories, Denise Riley, *War in the Nursery* (London: Virago, 1983).

58 D. Bardon, Y.I.M. Glaser, D. Prothero and D.H. Weston, ‘Mother and Baby Unit: Psychiatric Survey of 115 Cases’, *British Medical Journal*, 22 June 1968, 755–58, 756.

59 Natasha S. Mauthner, ‘“It’s a Woman’s Cry for Help”: A Relational Perspective on Postnatal Depression’, *Feminism & Psychology*, 1998, 8, 325–55.

60 Ibid., 326, 328.

61 Ann Oakley, *The Captured Womb: A History of the Medical Care of Pregnant Women* (Oxford: Basil Blackwood, 1984), 250.

62 Ann Oakley, *Women Confined: Towards a Sociology of Childbirth* (New York: Schocken, 1980).

63 Ibid., 146–47.

64 WC, SA/NCT/J/5/4, Information Sheets, NCT book-lets, photocopied articles, etc. 1980–90s, ‘Mothers Talking about Postnatal Depression’ (London: NCT, 1983), 25.

65 Ibid., Rachel, 15–16.

66 Lynn Abrams, ‘The Self and Self-Help: Women Pursuing Autonomy in Post-War Britain’, *Transactions of the Royal Historical Society*, 2019, 29, 201–21, 203.

67 Vivienne Welburn, *Postnatal Depression* (London: Fontana, 1980), 75.

68 Ibid.

69 Marilyn Wilson, ‘Breastfeeding on Demand’, *Spare Rib*, September 1981, 23.

70 Welburn, *Postnatal Depression*, 192–93.

71 Angela Phillips and Jill Rakusen, eds, *The New Our Bodies, Ourselves* (Harmondsworth: Penguin, 1978), 414, 420.

72 Diane Walters, ‘Postnatal Depression and Maternity Hospitals’, *Spare Rib*, May 1982, 22–23.

73 Margaret Dennis, *The First Weeks of Motherhood* (Oxford Post-Natal Support Group, The National Childbirth Trust, Handbook No. 2, December 1974), 4.

74 Ibid., 2.

75 Ibid., 4–5.

76 WC, SA/NCT/D/3/1, Postnatal Support within the NCT Papers, 1974–92, ‘Knowing my Limitations’, by Antoinette Ward, n.d. [c.1980–82]. See, for immature mothers and shifting ideas of responsibility, John Welshman, *From Transmitted Deprivation to Social Exclusion: Policy, Poverty and Parenting* (Bristol: Policy Press, 2009).

77 WC, SA/NCT/D/3/1, Postnatal Support within the NCT Papers, 1974–92, ‘Knowing my Limitations’, by Antoinette Ward, n.d. [c.1980–82].

78 Ibid.

79 WC, SA/NCT/D/3/3, NCT Papers on Postnatal Support and Postnatal Depression, NCT Postnatal Pack, Postnatal Depression Support 1987, 2 (emphasis in original).

80 Ibid., 2, 4.

81 Ibid., 11.

82 Ibid., 3.

83 Ibid., 30.

84 Ibid., 17.

85 WC, SA/NCT/J/2/2/1, Jean Curtis-Raleigh, ‘Depression after Childbirth’, *New Generation*, April 1968, 1:2, 8–9; Personal Account of a Mother, ‘Depression – An Illness Peculiar to Modern Society?’, *New Generation*, October 1968, 1:4, 4–5.

86 WC, SA/NCT/H/1/2/3, ‘Twenty-Five Years On’, 3.

87 WC, SA/NCT/M/1/5, NCT Press Cuttings: 1979–82, ‘Study Day on Post Natal Depression: Beating the New Baby Blues’ (publication not given), 4 June 1980.

88 WC, SA/NCT/J/2/2/3, ‘Postnatal Depression’, *New Generation*, September 1985, 4:3, 43–45, 43.

89 Ibid., 44.

90 WC, SA/NCT/J/2/2/2, ‘Postnatal Depression Workshop at Headquarters 21st November 1981’, *New Generation*, March 1982, 1:1, 24–5.

91 WC, SA/NCT/A/7/4, Hanna Corbishley’s papers and correspondence: Home birth 1976–89, Sheila Kitzinger, ‘Women’s Attitudes to the Place of Birth’. n.d. [c.1987].

92 Ibid., Appendix, ‘Some Women with Emotional Problems after Childbirth’, 37–38.

93 Sheila Kitzinger, *Homebirth: The Essential Guide to Giving Birth Outside of the Hospital* (London: Dorling Kindersley, 1991), 193.

94 Katharina Dalton, *Depression after Childbirth: How to Recognise and Treat Postnatal Illness* (Oxford and New York: Oxford University Press, 1980).

95 WC, SA/NCT/D/3/3, Postnatal Pack, Postnatal Depression Support 1987, 34.

96 John Cobb, *Babyshock* (London: Hutchinson, 1980), 118–19.

97 Welburn, *Postnatal Depression*; Carol Dix, *The New Mother Syndrome: Coping with Postnatal Stress and Depression* (London: Unwin, 1986); Anne Marie Sapsted, *Banish Post-Baby Blues: Advice, Support and Encouragement for Preventing and Coping with Postnatal Depression* (Wellingborough: Thorsons, 1990).

98 Kendell, ‘Emotional and Physical Factors’, 3.

99 WC, SA/NCT/A/7/1, Hanna Corbishley’s papers and correspondence: Postnatal Depression, 1971–83, D. Bardon, ‘Puerperal Depression’, typescript of paper appearing in *Midwife and Health Visitor*, January 1972, 3.

100 Ibid., various papers by D. Bardon; D. Bardon, ‘Psychological Implications of Provision for Childbirth’, *The Lancet*, 8 September 1973, 555–58, 557, 556.

101 WC, SA/NCT/A/7/1, D. Bardon, ‘Puerperal Sickness’, Read at a National Course for General Practitioners on Psychosomatic Medicine – Life Events and Disease, held at Central Middlesex Hospital on June 6, 1974, 2.

102 Ibid.

103 Ibid., A.A. Baker, *Psychiatric Disorders in Obstetrics* (Oxford: Blackwell, 1967), 16.

104 WC, SA/NCT/A/7/1, Bardon, ‘Puerperal Sickness’, 2; Bardon, ‘Puerperal Depression’, 2.

105 WC, SA/NCT/A/7/4, Letters from Desmond Bardon to ‘Ann’ [Bowen Jones, National Secretary to the NCT], 17 January 1981 and 24 January 1981.

106 WC, SA/NCT/A/7/1, D. Bardon, ‘Puerperal Depression’, *Psychosomatic Medicine in Obstetrics and Gynaecology*, 3rd International Congress, London 1971, 335–37, 5-page typescript.

107 Ibid., 1.

108 Jennifer Crane, *Child Protection in England, 1960–2000: Expertise, Experience and Emotion* (Cham: Palgrave Macmillan, 2018), 2.

109 WC, SA/NCT/J/2/2/1, Personal Account of a Mother, ‘Depression’, 5.

110 WC, SA/NCT/J/5/4, ‘Mothers Talking about Postnatal Depression’, Foreword.

111 WC, SA/NCT/J/5/4, ‘Mothers Talking about Postnatal Depression’. John Illman, ‘Why Mother Feels so Much Better after a Good Scream’, *Daily Mail*, 14 October 1983, 3. Illman highlighted the recommendation in the NCT booklet to scream and pound to get rid of pent up emotions and women’s own accounts and their advice involving diet, relaxation and support.

112 WC, SA/NCT/J/5/4, ‘Mothers Talking about Postnatal Depression’, Carol, 10.

113 Ibid., Margaret, 6.

114 Ibid., Ann, 8–9.

115 Ibid., Hilary, 5.

116 Ibid., Diane, 20–21.

117 WC, SA/NCT/J/2/2/2, ‘Postnatal Depression: How Does it Feel?’, 28.

118 Ibid., 28–29 (emphasis in original).

119 WC, SA/NCT/J/5/4, ‘Mothers Talking about Postnatal Depression’, Rachel, 15–16.

120 Ibid., Margaret, 6.

121 D.W. Winnicott, *Babies and their Mothers* (Reading, Mass: Addison-Wesley, 1987), 8–9. See Michal Shapira, *The War Inside: Psychoanalysis, Total War, and the Making of the Democratic Self in Postwar Britain* (Cambridge: Cambridge University Press, 2013), for the impact of psychoanalysis in the development of attachment theory and on ideas about the mother–child relationship in post-war Britain.

122 WC, SA/NCT/J/2/2/2, ‘Back to Basics: A Thought-Provoking Article on the Essence of Postnatal Support by Trust Advisor Desmond Bardon’, *New Generation*, March 1983, 2:1, 27–8, 28.

123 Davis, *Modern Motherhood*, 36–37.

124 Ibid., 37.

125 WC, SA/NCT/J/2/2/3, ‘Soapbox: Guilt, Failure and the NCT’, *New Generation*, December 1986, 5:4, 20–21.

126 Davis, *Modern Motherhood*, 73.

127 Ibid., 72–74.

128 Ibid., ch. 5.

129 A survey of Manchester NCT members in 1986 reported that 14 per cent found postnatal support unsatisfactory, 5 per cent unhelpful and 10 per cent non-existent: WC, SA/NCT, J/2/2/3, ‘The Quality of Support is Surveyed and PNC’, *New Generation*, June 1986, 89:1, 44–45, 45.

130 WC, SA/NCT/J/5/1, NCT booklets and leaflets, 1981–c.2000, ‘Postnatal Depression’ (NCT Publishing, 1990); WC, SA/NCT/E/5/3, NCT Policies and Briefings 1994–2001, The National Childbirth Trust 1956–66: 40 Years of Birth in Britain, no page nos: 61 per cent were educated to a least degree level, 71 per cent were professionals and 68 per cent regarded themselves as middle class.

131 WC, SA/NCT/J/5/4, ‘Mothers Talking about Postnatal Depression’, ‘Open Letter from Dr Bardon’, 27–29, 29.

132 Fiona Cust, ‘Peer Support for Mothers with Postnatal Depression: A Pilot Study’, *Community Practitioner*, January 2016, 89:1, 3841; Action on Postpartum Psychosis, ‘Peer Support for You’: https://www.app-network.org/peer-support/

